# Hyperhemolysis in a patient with β-thalassemia major

**DOI:** 10.4103/0973-6247.45259

**Published:** 2009-01

**Authors:** Lakmali R. Morawakage, B. J. C. Perera, P. D. N. Dias, S. K. Wijewardana

**Affiliations:** *Transfusion Medicine, National Blood Centre, Sri Lanka*; 1 *Lady Ridgeway Children Hospital, Sri Lanka*

**Keywords:** β-thalassemia, direct antiglobulin test, hyperhemolysis, transfusion

## Abstract

A case of hyperhemolysis in a 2-year-old boy with β thalassemia major was noted. After several transfusions, he developed hyperhemolysis with a positive (C3d only) direct antiglobulin test (DAT) and no clinically significant RBC allo- or auto-antibodies. (There was a weak cold antibody, showing a narrow thermal range). Because there was no significant improvement with steroid and immunoglobulin infusions, cyclophosphamide therapy was tried with notable success.

## Introduction

The term hyperhemolysis is applied when the post-transfusion hemoglobin (Hb) level is less than the pre-transfusion level, suggesting destruction of both the patient's own red blood cells (RBCs) and the transfused RBCs. The mechanism of hyperhemolysis is not well understood although it has been well described in patients with sickle cell disease. Transfusion of antigen-negative cross-match compatible RBCs does not prevent hyperhemolysis. Serologic studies of post-transfusion and follow-up samples may show a negative or positive (complement only) direct antiglobulin test and usually no RBC antibodies, leaving the hemolysis unexplained (i.e., there is no evidence of red cell antigen + antibody-mediated immune destruction). In a mild form, the option is to stop transfusion to avoid exacerbating the hemolysis. In severe forms, further transfusions have been given successfully only with concurrent IVIg and IV steroid therapy. Recurrent hyperhemolysis is fortunately rare and is unpredictable.

Awareness of the potential for recurrent hyperhemolysis is important because, where possible, alternative therapy should be sought and transfusion should be avoided. For patients with past history of recurrent hyperhemolysis, if transfusions are required, use of concurrent IVIg and steroids should be considered. Note, however, that because infusion of IVIg has been associated with renal toxicity, thrombosis and estimated 0.6% risk of stroke, its use should be selective.

## Case Report

A two-year-old Sri Lankan Tamil boy, diagnosed with β thalassemia major 6 months ago, was transferred to the tertiary care hospital for children for the management of severe anemia. His hemoglobin on arrival was 3.1 g/dl, even though he had received 600 ml of RBC transfusions on two consecutive days, five days ago. Six months earlier, when he was first diagnosed with β thalassemia, he was started on monthly RBC transfusions that lasted three months, and then the transfusion interval was gradually reduced to every few days due to very low pre-transfusion hemoglobin levels. 

At the time of transfer, the patient was severely pale and deeply icteric with a large spleen (8 cm) and liver (3 cm) but there were no signs of heart failure. The diagnosis of β thalassemia major was confirmed by high performance liquid chromatography (HPLC). Both parents were diagnosed as having the thalassemia trait. Transfusions with leukoreduced red cells matched for Rh and Kell antigens were given at 4-6 day intervals. The patient developed fever on and off but his clinical condition generally remained the same with high total serum bilirubin of 137 μmol/l with indirect serum bilirubin of 115 μmol/l. Coagulation screen, renal function tests and serum proteins were normal, and there was no evidence of G6PD deficiency or malarial infection. A blood culture was negative. A bone marrow biopsy showed a poorly managed major hemoglobinopathy with increased iron stores and no evidence of any storage diseases.

Serological investigations confirmed his blood group as O R_1_R_1_ with a phenotype of K-, k+, Fya+, Fyb+, Jka+, Jkb-, Lea-, Leb+, MMss. There was a weak cold autoantibody reactive only in undiluted serum in NISS below 20°C. The DAT was positive (2+) with C3d only. Unexpected antibodies were not detected in any of the serum samples. An eluate could not be done due to the low Hb level. 

Despite transfusion with cross-match compatible, leukoreduced and phenotype-selected blood in doses of 20 ml/kg, the patient continued to hemolyze. Oral prednisolone and azothioprin were given with no apparent improvement. Azothioprin was stopped due to elevated liver enzymes and the discovery of free fluid in the abdomen. Addition of intravenous immunoglobulin at 0.4 g/kg/day for 7 days showed no therapeutic benefit. High doses of cyclophosphamide therapy at 50 mg/kg were given in two doses, three days apart. During the cyclophosphamide therapy, the size of the spleen reduced to 4 cm, and the DAT became negative for C3d. A weakly reactive DAT with anti-IgG was noted, possibly attributable to immunoglobulin therapy. While receiving initial cyclophosphamide therapy, the patient developed a lower respiratory tract infection with positive radiological findings that was successfully treated with intravenous antibiotics. After the second dose of cyclophosphamide, the patient became more lethargic with Hb level of 2.7 g/dl. Transfusion of Rh and Kell phenotype-matched RBCs was given in a dose of 20 ml/kg. His post-transfusion Hb was 6 g/dl with no evidence of hyperhemolysis and an obviously improved clinical condition. Hemoglobin was around 6 g/dl for about three weeks and was followed by regular monthly transfusions.

## Discussion

A case of hyperhemolysis causing severe anemia in a patient with β thalasemia major, who continued to hemolyze despite use of leukoreduced and phenotype-matched transfusions was reported. He had no demonstrable clinically significant RBC antibodies at any time but showed a positive DAT with C3d only which disappeared after immunosuppressive therapy with cyclophosphamide. The exact pathogenesis of hyperhemolysis is complex and poorly understood. It involves destruction of both transfused and autologous RBCs.

Hyperhemolysis was first recognized in patients with sickle cell disease.[1–3] It is rarely reported in thalassemic patients.[4] Some theories that have been proposed based on observations principally of sickle cell patients who are chronically transfused include suppression of erythropoiesis, hyperactive macrophages causing bystander hemolysis, defective regulation of complement, and antibodies below the limit of detection of current serologic method, IgA antibodies or possibly HLA antigen-antibody reactions. In sickle cell disease patients, hyperhemolysis is commonly associated with reticulocytopenia, which may reflect suppression of erythropoiesis.[2] However, responses to steroids suggest something more than erythropoiesis suppression may be involved in its pathogenesis. In the absence of RBC antibody-mediated hemolysis, hyperactive macrophages could be responsible for destruction of the patient's own red cells as well as the transfused cross-match compatible cells.[3]

The RBCs of patients with sickle cell anemia have defective regulation of the complement membrane attack complex.[5] This defect may make sickle cells more susceptible to ‘bystander hemolysis” in which immune complexes cause lysis of bystander, antigen negative red cells.[1,2,6,7] In this case there is no detectable alloantibody. Red cell destruction can occur by antibody-dependent cell mediated cytotoxicity (ADCC) with levels of antibody that are below the serological detection threshold. Cell mediated lysis independent of antibody may occur for red cell antigens in the absence of detectable antibody.[8–10] Some red cells express HLA antigens and HLA antibodies and can cause red cell hemolysis.[11,12] Hemolysis due to HLA antigen and antibody reaction by hyperactive macrophages and bystander hemolysis of HLA antigen negative transfused cells could be another possible mechanism of red cell lysis with no detectable red cell alloantibody.[13] Incomplete IgA antibodies can produce hemolysis with negative findings using common serological techniques.[14] ^51^Chromium-labelled red blood cell survival studies, if available, should be considered whenever an unexplained hemolytic transfusion reaction occurs, or when an expected red blood cell alloantibody cannot be demonstrated by in vitro laboratory studies.[15]

This patient's dramatic response to cyclophosphamide suggests that cyclophosphamide can be used in patients with hyperhemolysis where transfusion is unavoidable due to other associated medical conditions.
